# The Classroom Discourse Observation Protocol (CDOP): A quantitative method for characterizing teacher discourse moves in undergraduate STEM learning environments

**DOI:** 10.1371/journal.pone.0219019

**Published:** 2019-07-17

**Authors:** Petra Kranzfelder, Jennifer L. Bankers-Fulbright, Marcos E. García-Ojeda, Marin Melloy, Sagal Mohammed, Abdi-Rizak M. Warfa

**Affiliations:** 1 Department of Biology Teaching and Learning, University of Minnesota-Twin Cities, Minneapolis, Minnesota, United States of America; 2 Molecular and Cellular Biology, University of California-Merced, Merced, California, United States of America; 3 Biology Department, Augsburg University, Minneapolis, Minnesota, United States of America; Murray State University, UNITED STATES

## Abstract

We describe the development and validation of a new instrument, the Classroom Discourse Observation Protocol (CDOP), which quantifies teacher discourse moves (TDMs) from observational data in undergraduate STEM classrooms. TDMs can be conceptualized as epistemic tools that can mediate classroom discussions. Through an inductive–deductive coding process, we identified commonly occurring TDMs among a group of biology instructors (*n* = 13, 37 class session) teaching in Active Learning Environments. We describe the CDOP coding scheme and its associated matrix that allows observers to reliably characterize TDMs in 2-min time intervals over the course of a class period. We present the protocol, discuss how it differs from existing classroom observation protocols, and describe the process by which it was developed and validated. Also, we show how this protocol is able to discriminate the discursive practices of instructors teaching in undergraduate STEM learning environments with sample qualitative and quantitative results that illustrate its utility for assessing and improving STEM instructional practices.

## Introduction

Active learning strategies are broadly defined as activities that increase student engagement in learning processes [1,2]. Recent efforts in undergraduate science, technology, engineering, and mathematics (STEM) education reform emphasize the need for documenting the degree to which these active learning strategies are used in undergraduate learning environments [3–5]. Yet, beyond comparative studies of how effective active-engagement instruction is in relation to traditional instruction (e.g., [6]), little is known about *how* the new changes promote undergraduate STEM learning [7]. One possibility is that active-engagement instruction increases the frequency of classroom interactions (e.g., whole, group-based, and one-on-one discussions), creating opportunities for pedagogically rich classroom discourse, including teachers’ use of general conversational strategies and specific discourse practices that might improve student understanding of content knowledge [8, 9]. However, beyond noting increases in the frequencies of classroom interactions and behaviors via classroom observations [10–12], the nature of teacher-initiated discourse moves (TDMs) and how such practices are orchestrated remain understudied [3]. One reason for this is the lack of appropriate instruments that can measure the nature of TDMs in a valid and reliable manner. Therefore, we sought to develop and validate a new instrument, called the Classroom Discourse Observation Protocol (CDOP), to reliably quantify TDMs in undergraduate STEM learning environments.

### Theoretical background

#### Teacher discourse moves (TDMs)

TDMs can be conceptualized as epistemic tools that can mediate classroom discussions [13]. With these discourse moves, the instructor engages students in the construction, justification, and evaluation of knowledge as opposed to simply providing factual knowledge [14, 15]. Similarly, Ohlsson [16] operationalized TDMs as actions which function to promote the creation and development of knowledge and understanding. Thus, TDMs can be thought off as mechanisms for promoting student thinking and generation of knowledge.

Extensive work has been done on TDMs in mainly primary and secondary STEM classrooms, especially mathematics [17–19]. These studies suggest that the Initiate-Response-Evaluate (IRE) discourse pattern, which focuses on fixed transmission of unchanged ideas and allows little opportunity for collaborative talk, remains the pedagogical default in STEM instruction (see reviews by [9,20]). This is in contrast to more dialogical approaches, such as the Initiate-Response-Feedback (IRF) discourse pattern, which focuses on creating opportunities for dialogue with students by allowing different voices to be heard, generating collaborative discussions, and cumulatively building on students’ ideas [21, 22]. Dialogical discourse approaches, such as IRF, are assumed to be the most effective in promoting student learning of scientific ideas [15, 23], and Duschl [14] specifically argues that instruction in science education should focus on engaging students in the “dialogic knowledge-building processes that are at the core of science” (p. 269).

The emphasis on dialogical discourse patterns that promote student engagement raises important questions, such as how to identify discourse practices and measure them in a valid and reliable manner. Additionally, in response to national efforts aimed at improving undergraduate STEM education, there has been an explosion of Student-Centered Active Learning Environment for Undergraduate Programs (SCALE-UP) or Active Learning Classrooms (ALCs) at many universities and colleges across the globe. ALCs are defined as learning spaces in which the learner is actively engaged and the spaces have been optimized for higher student interactions with their peers and the instructor [2]. In these new learning environments, there is an increased likelihood of dialogical discourse patterns happening; therefore, there is a need to both understand the nature of classroom interactions and to quantify the discourse patterns happening in undergraduate STEM learning environments.

### Current tools for observing student-teacher interactions

To date, most observational protocols used in undergraduate STEM learning environments focused on characterizing the active-engagement nature of classroom instruction [5, 10, 24, 25]. However, two approaches are commonly used to analyze TDMs. The first approach uses qualitative coding of teacher-student interactions in which observers must describe and thematically code the teaching observed in the episodes [26–29]. A problem with this approach is that they often rely on unstructured rubrics to codify the observed behaviors. The second approach relies on global ratings using Likert scale questions (e.g., [24]). The classroom observation protocols using Likert scale questions, such as the Reformed Teaching Observation Protocol (RTOP), are useful for giving us an overall view of classroom practices and capture coarse measures of classroom instruction, but are not designed to capture the dynamic nature of classroom discourse [24].

Recently, the education research community has developed a newer set of classroom observation protocols, such as the Teaching Dimensions Observation Protocol (TDOP) [5] and Classroom Observation Protocol for Undergraduate STEM (COPUS) [10], that can be used to describe instructor and student classroom behaviors. While COPUS and other extant protocols can measure the prevalence of engaged instruction in undergraduate STEM learning environments, they do not provide a way to measure TDMs. Thus, there is a need for an instrument that can reliably measure and analyze the nature of classroom discourse. Such instrument would be used in conjunction with tools, such as the RTOP, TDOP, and COPUS, to better capture the impact of instructional practices on student learning in undergraduate STEM learning environments.

### Study objectives

The goal of this study was to develop an instrument, the CDOP, which can quantify TDMs from observational data in undergraduate STEM learning environments. Therefore, the two major objectives were to:

- Objective 1: Identify a set of codes that capture commonly observed TDMs.
- Objective 2: Develop a quantitative method based on a 2-minute time period that captures the occurrences and the dynamics of TDMs over the course of a class period.

## Development of CDOP

### Study context

We evaluated thirteen faculty teaching mostly introductory undergraduate biology courses (majors and non-majors) in Active Learning Environments (ALEs) at a large Midwestern research-intensive institution in the United States. ALEs are defined as including the following three factors: 1) ALCs, which are defined as learning spaces in which the learner is actively engaged and the spaces have been optimized for higher student interactions with their peers and instructor [2]; 2) leadership (i.e. college dean and department head) that values active-engagement instruction and discourages exclusive use of traditional lectures; and 3) faculty training in evidence-based scientific teaching practices (e.g. Summer Institutes on Scientific Teaching). Twelve of the 13 faculty we studied taught in ALCs, while one faculty taught in a traditional lecture classroom with theater-like seating. Table 1 provides detailed characteristics of the faculty members and courses involved in this study. The study was approved by the Human Subjects Committee of the University of Minnesota’s Institutional Review Board (Study Number: STUDY00000896).

**Table 1:** Demographic characteristics of faculty members (*n* = 13)^a^ and their courses (37 class sessions).

Characteristics	*N*
Years of faculty experience	
0–5	2
5–10	4
>10	7
Faculty appointment line	
Tenured/Tenure track	6
Teaching Assistant/Associate	7
Classroom type	
Traditional lecture classroom	1
Active learning classroom	12
Number of instructors per course	
1	4
2	9
Number of students per course	
30–65	1
100–135	10
135–170	1
170–205	1
Student type	
Biology majors	7
Biology non-majors	6
Class level	
1000–2000 (lower division)	11
>3000 (upper division)	2

^a^Total number of faculty is 13, but one faculty member is affiliated with all three departments and counted accordingly.

### Classroom video recordings

We conducted classroom visits and video recordings using a Swivl™ C Series Robot with five remote markers (microphones) and an Apple iPad. The Swivl™ robot rotates to follow the marker worn by the instructor and captures audio as well as video. The other four markers are placed randomly on students’ tables to record audio. We made classroom visits on a weekly or biweekly basis to ensure that we didn’t observe any changes in the instructor’s teaching practices over the course of the semester. To capture instructors’ representative teaching practices, we video-recorded two or three class sessions for each instructor during the middle of the semester. Class sessions ranged from 50 to 115 minutes, and we avoided class sessions where the entire meeting time was dedicated to exams, student presentations, or special group project work for recordings, because these situations would limit the diversity of discourse moves used by the instructors. However, we included class sessions in which quizzes were given since these are a regular part of the weekly class sessions and only took 30–45 minutes of the class session (quizzes were often taken individually, discussed and completed again as a group for credit).

### Classroom observation protocol for undergraduate STEM data collection

We used COPUS [10] to characterize the classroom behaviors of our 13 instructors and their students, reaching a high average inter-rater reliability (IRR) between coder pairs (κ = 0.79). Based on these COPUS data, we selected one class session from each of the six instructors that had the highest average collapsed codes, instructor *guiding* and student *talking to class*, for verbatim transcription and development of our CDOP coding scheme. As described in Smith, Vinson [11], *guiding* is a collapsed COPUS instructor code that contains the following six individual codes: *listening to and answering student questions to entire class*, *asking clicker question*, *follow-up/feedback on clicker question or activity*, *moving through class guiding ongoing student work*, *posing non-clicker question to students*, and *one-on-one extended discussion with individual students*, while *student talking to class* is a collapsed COPUS student code that contains the following four individual codes: *student answering question posed by instructor*, *student asks question*, *students engaged in whole-class discussion*, and *students presenting to entire class*. We selected class sessions with a high prevalence of the COPUS collapsed codes *guiding* and *student talking to class* since they indicate class sessions where the instructor teaches using active learning strategies, creating more opportunities for classroom interactions that might lead to more opportunities for pedagogically rich classroom discourse.

### CDOP coding scheme: Qualitative coding of class transcripts

**Fig 1:**
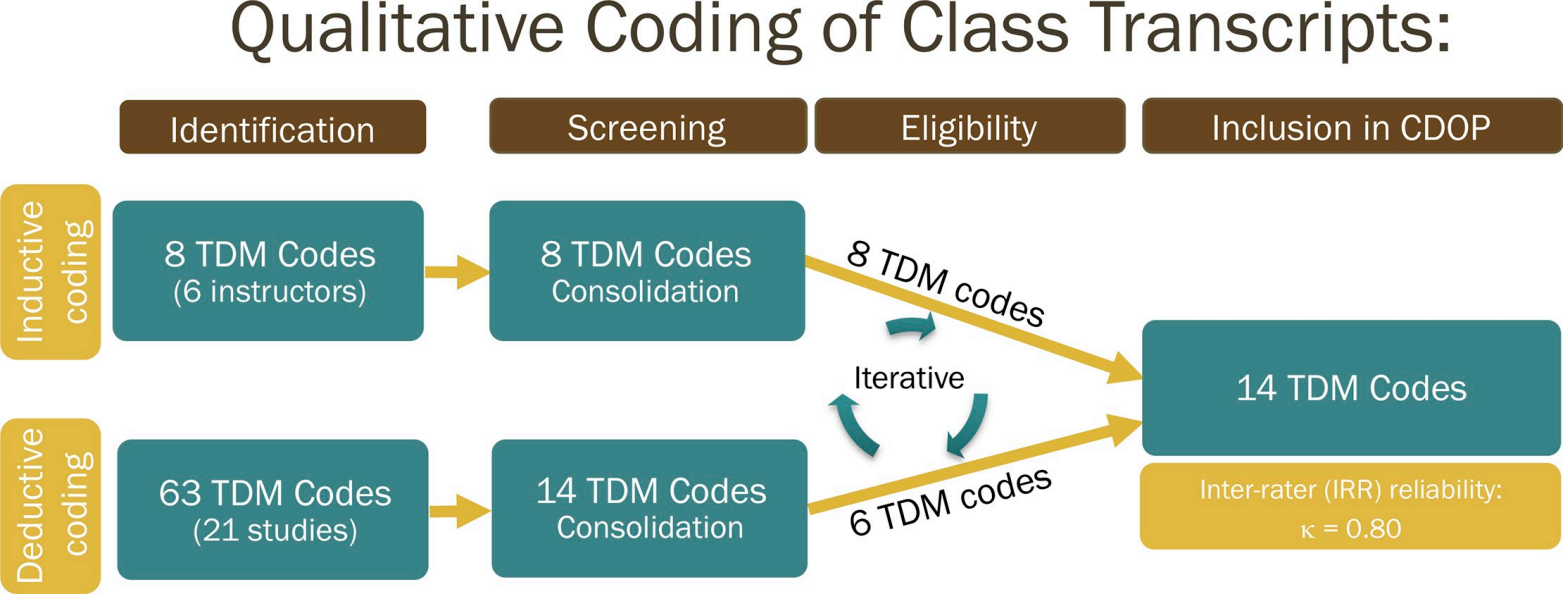
Flowchart of qualitative coding of class transcripts to develop CDOP coding scheme.

A major goal of our study was to create a protocol for identifying and categorizing TDMs, and training coders to use this protocol. Our immediate goal was to find a coding scheme that would be both comprehensive and efficient but would also enable observers to reliably analyze instructor discourse moves. To this end, we developed the CDOP codes by identifying TDMs through an iterative process of deductive and inductive coding approaches (Fig 1). We started by using a comprehensive literature search to identify extant protocols for categorizing our target variable (TDMs) and a grounded theory approach [30] for identifying emergent codes from our own data. In all of our analyses, we selected episodes of classroom interactions (e.g., whole, group-based, and one-on-one discussions) that we divided into instructor and student dialogue turns as the primary source of data.

#### Literature-based codes

Several studies have examined instructional discourse moves in the context of secondary schools. For example, Pimentel and McNeill [31] investigated secondary school science teachers’ approaches to discussion during the piloting of an urban ecology curriculum designed to support student participation in science discourse. They identified four categories of teacher moves to code classroom discourse: teacher elaboration, cutoff, probing, and toss back. Some of these moves were similarly identified by Michaels and O’Connor [22] who have extensively studied TDMs. Other researchers, such as Chin [32], focused on teachers’ questioning and probes as a means to stimulate productive thinking. As shown in Fig 1, we adapted these extant coding schemes to describe the discourse behavior of our instructors. Overall, we used 23 peer-reviewed observational studies of TDMs from secondary or undergraduate STEM classrooms to categorize the discourse behavior of our observed instructors. Table 2 shows a list of codes, code sources, code descriptions, and example dialogues that our coding scheme adapted from previous work.

**Table 2:** CDOP coding scheme.

**1. Teacher-centered: Instructor is talking about content**			
**Codes**	**Code Source**^**a**^	**Code Description**	**Example Dialogue**^**b**^
**Evaluating**	Hardman [33], Warfa, Roehrig [34], Rasmussen, Kwon [35], Sinclair and Coulthard [36], Mehan [37], Garton [38], Chin [32]	Instructor repeats, accepts and/or rejects student's response, or acknowledges that they don't know the answer to a student's question.	Student: Then you multiply those together and get the probability by dividing the number of fertilization events. **Instructor: Total fertilization events. Okay.**
**Forecasting**	Current study	Instructor associates current topics to future topic.	**Instructor: You're going to do something in lab actually focused on human population and population growth.**
**Linking**	Current study	Instructor associates past topic to current topic.	Student: You don't have a bigger potential as well because there's more connections, there's more access to the axon terminals? **Instructor: Well, remember, we had that summation of action potentials. We had an action potential and we had the nodes and it could split off.**
**Real-worlding**	Current study	Instructor relates ideas to conventional knowledge, broader perspective, and instructor’s or student's personal experiences.	**Instructor: Successful genotypes-look around the room. Nothing but winner in this room, right? We have all made it to reproductive age.**
**Sharing**	Warfa, Roehrig [34], Krussel, Edwards [39], Pimentel and McNeill [31]	Instructor shares information, answers student question, or provides instructions for finding the solution.	**Instructor: Just think of, kind of, chromatid pairs, sister chromatid paired, it’s a little easier to think of the numbers.**
**2. Student-centered: Instructor asks students to talk about content**			
**Codes**	**Code Source**^**a**^	**Code Description**	**Example Dialogue**
**Generative**	Warfa, Roehrig [34], Lidar, Lundqvist [40], Criswell and Rushton [41], Chin [32]	Instructor asks student to recall facts, and basic concepts, or related information.	**Instructor: Those come together in fertilization to make a zygote, right?** Student: Yes.
**Checking-in**		Instructor asks student if they have a question or need clarification.	**Instructor: Does that make sense?; Do you have any questions?; How's it going?; Are we good?**
**Clarifying**	Herbel-Eisenmann, Steele [19], O'Connor, Michaels [23], MacDonald, Miller [42], Chin [32]	Instructor asks student to elaborate on condensed, cryptic, or inexplicit statement.	**Instructor: Can you say more about that? What do you mean by that? Can you give an example?**
**Connecting**	Current study	Instructor asks student to associate past topic to current topic.	**Instructor: Costs of sex that haven't been mentioned plus what we've been talking about for the last week.** Student: Is it overpopulation?
**Contextualizing**	Herbel-Eisenmann, Steele [19], Krussel, Edwards [39], Criswell and Rushton [41]	Instructor asks students to connect ideas to conventional knowledge, broader perspective, and their personal experiences.	**Instructor: Anyone have an example that they really want to hear about/talk about (referring to student responses to finding analogies between cell processes and common household items)?**
**Representing**	Current study	Instructor asks student to create a visual or mathematical representation of content.	**Instructor: Think about how you could draw that out, too.**
**Constructing**	Criswell and Rushton [41], NGSS Lead States [43]	Instructor asks students to build knowledge by interpreting and/or making judgments based on evidence, data, and/or model.	**Instructor: In your own words, what is your conclusion when you look at those data?**
**Requesting**	O'Connor, Michaels [23], Rasmussen, Kwon [35], MacDonald, Miller [42]	Instructor asks student to justify or explain their reasoning.	**Instructor: I’m liking what I see but explain it to me** (referring to student whiteboard work calculating the number of fertilization events that produce a specific offspring).
**Explaining**	Current study	Instructor asks student to explain reasoning to other students.	**Instructor: Can you explain your work to everybody else at your table so that they can figure that out?**
**Challenging**	Michaels and O’Connor [22], O'Connor, Michaels [23], O’Connor, Michaels [44]	Instructor asks student to evaluate another student's idea.	**Instructor: Cost of sex?** Student: Pregnancy. **Instructor: I acknowledge that it's a good point, and why is there a problem with calling pregnancy a cost evolutionarily?**
**3. Other**			
**Codes**	**Code Source**^**a**^	**Code Description**	
**No content discourse**	Seidel, Reggi [45]	Instructor is not talking or asking students to talk about content.	
**Other**	Current study	TDM not described by these codes.	

^a^Sources of the deductive codes were 23 peer-reviewed, observation-based studies of TDMs from secondary or undergraduate STEM classrooms (see reference list). The inductive codes (current study) were those that emerged from our coding of class transcripts and videos using the Strauss and Corbin [30] grounded theory approach.

^b^The instructor portion of the dialogue associated with the CDOP code is shown in bold font. The student portion of the dialogue is shown for context.

#### Emergent, inductive codes

When the extant codes in the literature did not capture a discourse behavior we observed, then we used an inductive approach to identify and categorize the target behaviors. That is, we used the grounded theory approach developed by Strauss and Corbin [30] to identify codes that were emergent from our coding of classroom transcripts and videos. This involved an iterative process of coding and recoding until we established a high inter-rater reliability (IRR) between coder pairs as described later in the paper.

Our coding scheme went through nine different versions of field-testing and feedback between all members of the research team and formed the basis of the CDOP coding scheme shown in Table 2. In addition to the codes, the source of the codes, and the coding description, we showed dialogue examples drawn from our study that fit within each coding category in Table 2.

### CDOP coding matrix: Quantitative coding of audio class recordings

**Fig 2:**
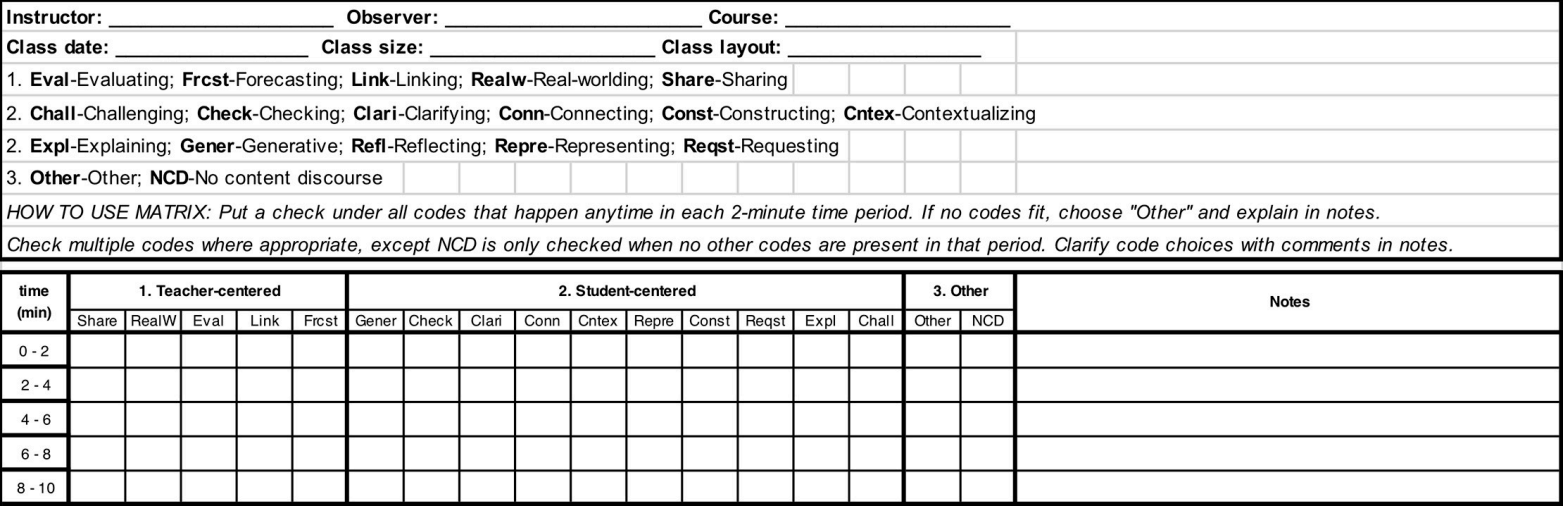
An excerpt of the CDOP matrix.

We coded the six audio class recordings using the CDOP coding scheme (Table 2) and the CDOP matrix (Fig 2). The CDOP matrix allowed observers to measure TDMs at 2-minute time periods, checking all TDM codes that occurred within a single 2-minute time period with one exception: *no content discourse* was only checked if no other TDM codes had been checked in that 2-minute time period (i.e., the instructor did not talk about content during that entire time period). If a TDM was observed, but no identified codes fit, then the observer chose *other* and described the new TDM in the notes section. In general, all code choices were clarified with comments in the notes section.

After we completed the development work with the six top instructors, we further tested the CDOP coding scheme and matrix by including audio recordings of the remaining seven instructors in our study until we established high IRR between coder pairs. During this process of internal validation, we added two additional codes, *constructing* and *contextualizing*, to the CDOP coding scheme (Table 2).

#### Analyzing CDOP data

One of our main goals in developing the CDOP was to make a tool that could quantify TDMs of instructors teaching across all undergraduate STEM classrooms. Existing classroom observation protocols, such as COPUS, provide a descriptive tally of instructor behaviors (i.e., what an instructor is doing), but not the nature of their classroom discourse. The CDOP matrix data can be analyzed similarly to data obtained from the TDOP [5] and COPUS [10]. If a code is checked in a box, then it is replaced by the number *1*. If no code is checked in a box, then it is replaced by the number *0*. Then, these data can be used to identify how much time an instructor spends on different discourse moves over the course of a class period. To analyze the prevalence of different codes across class sessions, we added up how often each code was checked by the observer and then divided by the total number of codes similar to Smith, Jones [10]. For example, if an observer checks *sharing* 17 times out of a total of 35 codes in a class period, then the percentage of *sharing* happening in this class session would be 17/35, or 48.6% of the total codes.

We analyzed the COPUS and CDOP data for three instructors (A, B, and C) teaching in undergraduate STEM classrooms to show how the CDOP can be applied. In comparing the COPUS and CDOP results, our goal was to show how COPUS measures discourse behavior and CDOP measures student-instructor interactions, but not how the results of these instructors varied. First, we compared the COPUS instructor collapsed codes [11] to the CDOP collapsed codes of two instructors (A and B) teaching in ALEs. Second, we compared the COPUS instructor collapsed codes to the CDOP individual codes for an instructor (C) teaching in a non-ALC, and therefore, a limited ALE. However, interpretation of these results requires caution as we only analyzed these instructors to provide as a proof of concept. We plan to present a more in-depth analysis of how patterns of discourse behaviors vary across biology instructors teaching in undergraduate STEM classrooms in a future publication.

#### Reliability and validity

We established reliability between coder pairs using the CDOP in a two-stage process: 1) the IRR between coder pairs doing qualitative coding of class transcripts (*n* = 6) and 2) the IRR between coder pairs doing quantitative coding of audio class recordings (*n* = 13). We calculated IRR scores among coder pairs by calculating Cohen’s Kappa using the R package ‘irr’ [46]. Kappa scores in the range of 0.60–0.80 indicate substantial to high agreement according to Landis and Koch [47]. If Kappa scores were less than 0.60, then we conducted further training until all research members were using the codes in a similar manner, as indicated by Kappa scores in the range of 0.60–0.80.

In addition to establishing reliability for the CDOP, we conducted two commonly measured validity tests of new research instruments: face and content validity. Face validity is defined as reflecting the extent to which a measure reflects what it is intended to measure, while content validity measures how well an instrument assesses the construct of interest [48, 49]. In the context of the CDOP, face validity means examining whether a code adequately measures TDMs, whereas content validity means examining the clarity, operational definitions of the discourse moves, and overall structure of the CDOP. Both of these validity tests are commonly referred to as measures of internal validity. The most common way to establish internal validity is to ask a panel of experts to examine the instrument items and make judgements on whether they satisfy measures of face and content validity [50]. For this study, we asked a panel of experts with STEM disciplinary and education backgrounds (three PhDs and two PhD students at two research-intensive universities in the United States) to verify whether or not they thought that the CDOP measures TDMs and the degree to which the CDOP codes matched specifications of teacher discourse behaviors. More specifically, the panel received the CDOP coding scheme (Table 2) and the CDOP matrix (Fig 2) and were asked to evaluate four things: (1) representativeness of the content domain; (2) clarity of the codes and overall structure of CDOP; (3) usefulness of the tool for measuring TDMs; and (4) comprehensiveness of the CDOP [50].

To make the internal validity assessment more concrete, we calculated a Content Validity Index (CVI) score [50, 51] as follows: 1) all the panelists were asked to rate items related to validity on a strongly agee—strongly disagree Likert scale [1 = strongly disagree, 2 = neutral, 3 = strongly agree]; 2) we computed an average CVI score by dividing the number of expert agreements in a given category by the number of panelists (*n* = 5). The result (shown as a proportion) indicates the degree to which the expert panel agrees with each other on the validity of the CDOP. Davis (51) and Rubio, Berg-Weger (50) recommended a CVI score of 0.80 as a cutoff for new tools.

## Results

### The CDOP coding scheme

The CDOP coding scheme consists of 17 codes: 15 TDM codes that we developed through an iterative process of inductive and deductive coding (Fig 1), one code for documenting new TDMs (*other*), and one code for documenting when no or non-content discourse is taking place (*no content discourse*) (Table 2). We organized the coding scheme by how observers will code observations in undergraduate STEM classrooms and classified the codes broadly into teacher-centric and student-centric groups. The first five of the 15 TDM codes (*sharing*, *real-worlding*, *evaluating*, *linking*, and *forecasting*) are teacher-centric in that the dominant voice in the discourse belongs to an instructor mainly talking about content. For example, the code *sharing* connotes instructor discourse behavior in which an instructor shares content information with students, answers student questions, or provides instructions for finding a solution. Therefore, *sharing* involves telling, and ultimately, signifies direct instruction. Similarly, the code *evaluating* is teacher-centric in that it connotes instructor discourse behavior in which an instructor repeats, accepts and/or rejects a student's response, or acknowledges that they do not know the answer to a student's question (Table 2). Thus, this code is used to describe the “E” in the commonly reported IRE discourse pattern [38], which occurs after a student responds to a question initiated by the instructor.

The rest of the 15 CDOP codes are student-centric in that these codes reflect TDMs in which an instructor asks students to talk about content. The 10 codes are: *generative*, *checking-in*, *clarifying*, *connecting*, *contextualizing*, *representing*, *constructing*, *requesting*, *explaining*, and *challenging* (Table 2). For example, *generative* involves the instructor asking the student to recall facts and basic concepts or related information (e.g., [34]), *constructing* involves the instructor asking students to build knowledge by interpreting and/or making judgments based on evidence, data, and/or a model (e.g., [41]), and *requesting* involves an instructor asking students to justify or explain their reasoning [42]. The common denominator in all of these TDMs is that they involve students talking about the content (Table 2).

Below are excerpts of instructor and student discourse to illustrate the CDOP coding scheme and show its utility for identifying aspects of classroom discourse.

#### Instructor A: Sample excerpt containing teacher-centered and student-centered codes

In the excerpt below, CDOP codes are in parentheses and bold type. Students have been instructed to work in small groups on a worksheet that is introducing hematocrits (the ratio of the volume of red blood cells to the total volume of a blood).

1.1 Instructor: How are you guys doing? (**Checking-in**)
1.2 Student: Good. Well, I don’t really know the steps.
1.3 Instructor: Okay. Well you can look at them. So, what’s happening in the first picture? (**Generative**)
1.4 Student: Kind of just giving the blood draw.
1.5 Instructor: He’s just getting a blood draw. (**Evaluating**) Second picture? (**Generative**)
1.6 Student: I didn’t know if you wanted us to be more specific.
1.7 Instructor: Yeah, it can just be that. So, first you get some sample taken, (**Sharing**) then what is the point of this step? (**Generative**)
1.8 Student: Just to separate all parts of the blood.
1.9 Instructor: Yeah, (**Evaluating**) we're just separating it based on weight and then basically, we're measuring how much of each part we have. (**Sharing**)

The dialogue shown in this excerpt above contains two teacher-centric codes, *evaluating*, and *sharing*, and two student-centric codes, *checking-in* and *generative*. *Checking-in* (line 1.1: “How are you guys doing?”) is operationalized in the CDOP as a TDM in which an instructor asks students if they have questions or need a clarification (Table 2). This was a routine move that we observed the instructors use during small group instruction. We consider this student-centric in the sense that the instructor asks students if they need help understanding content. The other student-centric TDM in this dialogue–*generative*–is shown in lines 1.3, 1.5, and 1.7. In line 1.3, the instructor asks, “So, what’s happening in the first picture?” This forces students to talk about the content of the picture (line 1.4: “Kind of just giving the blood draw.”) and relate or recall information about the content. Thus, as operationalized in the CDOP, the purpose of a *generative* move is to force students to recall facts, basic concepts, or related content information (Table 2).

In contrast to the student-centric moves, teacher-centric TDMs in the CDOP focus on teacher acts. For instance, in lines 1.5 and 1.9, we see the instructor evaluating student responses either by repeating what the student said (line 1.5: “He’s just getting a blood draw.”) or with a simple agreement of yes (line 1.9: “Yeah.”). In an *evaluating* move, such as shown in lines 1.5 and 1.9, an instructor repeats, accepts, or rejects student responses or simply acknowledges they do not know the answer to a students’ question. This discourse move is as a means to assess student understanding of a concept or confirm the correctness of their response. For example, the simple utterance of “Yeah” in line 1.9 confirms the correctness of the student response in (line 1.8: “Just to separate all parts of the blood.”). In this instance, we see the instructor followed the *evaluative* move in line 1.9 by sharing with the student information related to how that separation is achieved (“we're just separating it based on weight and then basically, we're measuring how much of each part we have.”). We code all moves in which an instructor relays content information to students as *sharing*. *Real-worlding* is the other CDOP code that involves an instructor sharing content information; but it is differentiated from *sharing* since by using this move the instructor relates ideas to conventional knowledge, broader perspectives, and/or personal experiences (Table 2).

#### Instructor B: Sample excerpt containing mostly student-centered codes

In the excerpt below, the CDOP codes are in parentheses and bold type. Students have been instructed to open an online worksheet and work in small groups to create a logical/mathematical rule for determining the number of unique fertilization events that will produce a specific genotype in the offspring.

2.1 Instructor: Explain to me what you did. (**Requesting**)
2.2 Student 1: So, essentially, in each case, this is first column and for heterozygous possibilities. Essentially, we saw how many different combinations for the square genes we can get the right allele combination and how many times we can possibly get the right circle combination? Then you multiply those together and get the probability by dividing the number of fertilization events.
2.3 Instructor: Total fertilization events. Okay. (**Evaluating**) And how did you do it for this one? (**Requesting**)
2.4 Student 1: Well, this one is similar, except that there’s only one possibility for each because in each case there’s a homozygous which provides the same allele every time, and then there’s only one—there’s a possible combination to make a black, white and black, white, so there’s one times one. There’s only one out of four, and four’s the number that we got—four’s the number of fertilization events we got based on the fact that only two of these have two gene choices, and these are just one so to speak.
2.5 Instructor: And which genotype were you looking for? Was it the double heterozygous? (**Clarifying**)
2.6 Student 1: Yes, double heterozygous.
2.7 Instructor: Okay. Good. (**Evaluating**) In both cases? (**Clarifying**)
2.8 Student 1: This one, yes. Double heterozygous.
2.9 Instructor: Okay. Do you agree (to Student 2)? Does that make sense? (**Challenging**)

This instructor used mostly student-centric CDOP codes, including *requesting* (lines 2.1 and 2.3), *clarifying* (lines 2.5 and 2.7), and *challenging* (line 2.9) along with one teacher-centric CDOP code (*evaluating*). In the first move of this excerpt, the instructor asks students to explain their reasoning (line 2.1, *requesting*) and a student responds by explaining how they calculated the number of fertilization events. Next, the instructor evaluates the student response (line 2.3: “Total fertilization events.”) and confirms their reasoning with a simple “okay” and makes a second *requesting* move for another problem (line 2.3: “And how did you do for this one?”). In line 2.5, we see a *clarifying* move (“Which genotype were you looking for? Was it the double heterozygous?”), which is described in the CDOP as a move asking students to elaborate on condensed, cryptic, or inexplicit statement. The other student-centric CDOP code shown in line 2.9 is *challenging*. This TDM describes an instructor asking a student to evaluate another student’s idea, which is exemplified when the instructor asks a second student in the small group, “Do you agree? Does that make sense?” *Challenging* represents a TDM where an instructor asks students to go to another level with their content knowledge by engaging with others’ reasoning (Table 2).

#### Sample CDOP matrix data

**Fig 3:**
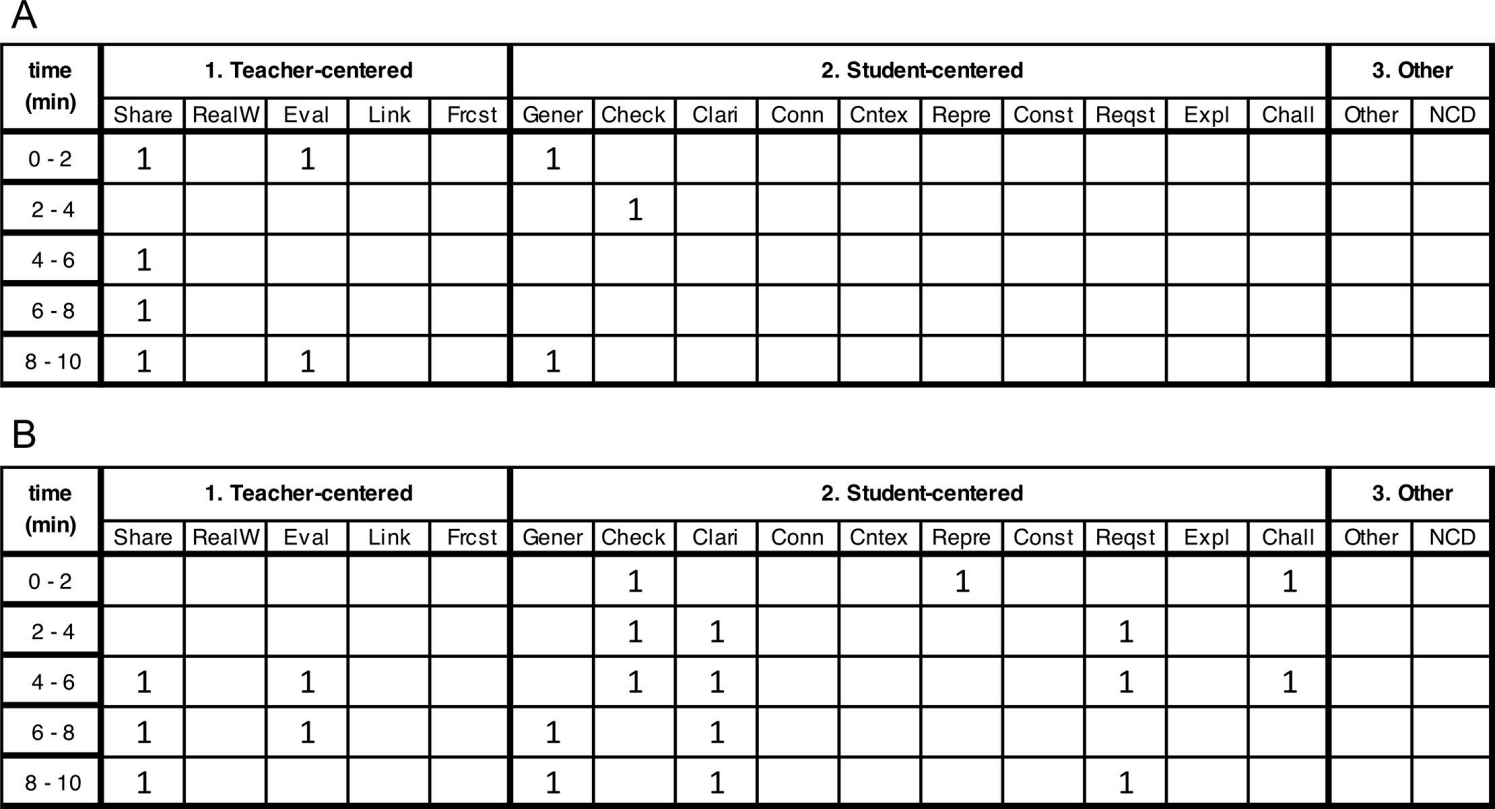
Examples of CDOP matrices with mostly *teacher-centered* TDM codes (A) and mostly *student-centered* TDM codes (B).

The analysis conducted on the sample excerpts above relied on qualitative coding of classroom transcripts, highlighting the development process of the CDOP coding scheme. However, our major objective in developing the CDOP was to make the coding process more quantitative in nature while still providing a descriptive account of the TDMs. To do this we created the CDOP matrix, modelled after the matrix used for the COPUS classroom observation protocol [10], which allows an observer to document all TDMs occurring within each 2-minute period over the length of a class session (Fig 2). The codes are grouped into *teacher-centered*, *student-centered*, and *other* and are arranged to facilitate the live coding of a class session. We used the CDOP matrix to code one audio recording from a class session for each of the two instructors (A and B) described above in the sample excerpts. Next, we used this coding to quantify the TDMs used by each instructor during the 10-minute period surrounding their sample excerpt. The CDOP matrix of the class session with more *teacher-centered* TDMs indicated that Instructor A used two *teacher-centered* TDMs (*sharing* and *evaluating*) and two *student-centered* TDMs (*generative* and *checking-in*) over the 10-minute period (Fig 3A). However, *teacher-centered* TDMs were used twice as often (6x) as the *student-centered* TDM (3x). In contrast, the CDOP matrix of the class session with more *student-centered* TDMs showed that Instructor B used the same two *teacher-centered* TDMs (*sharing* and *evaluating*), but used more *student-centered* TDMs (*generative*, *checking-in*, *clarifying*, *representing*, *requesting*, and *challenging*) (Fig 3B). Additionally, *teacher-centered* TDMs were used ¼ as often (5x) as *student-centered* TDMs (15x). Thus, this preliminary analysis demonstrates that the CDOP matrix provides a structured mechanism for identifying what discourse in happening in a class and documenting the frequency of usage of particular TDMs. These two examples illustrate how the CDOP matrix can be utilized to determine the TDMs used by an instructor without spending time subjectively evaluating what type of a discourse that is happening in an undergraduate STEM classroom.

### Comparison of COPUS and CDOP results

**Fig 4:**
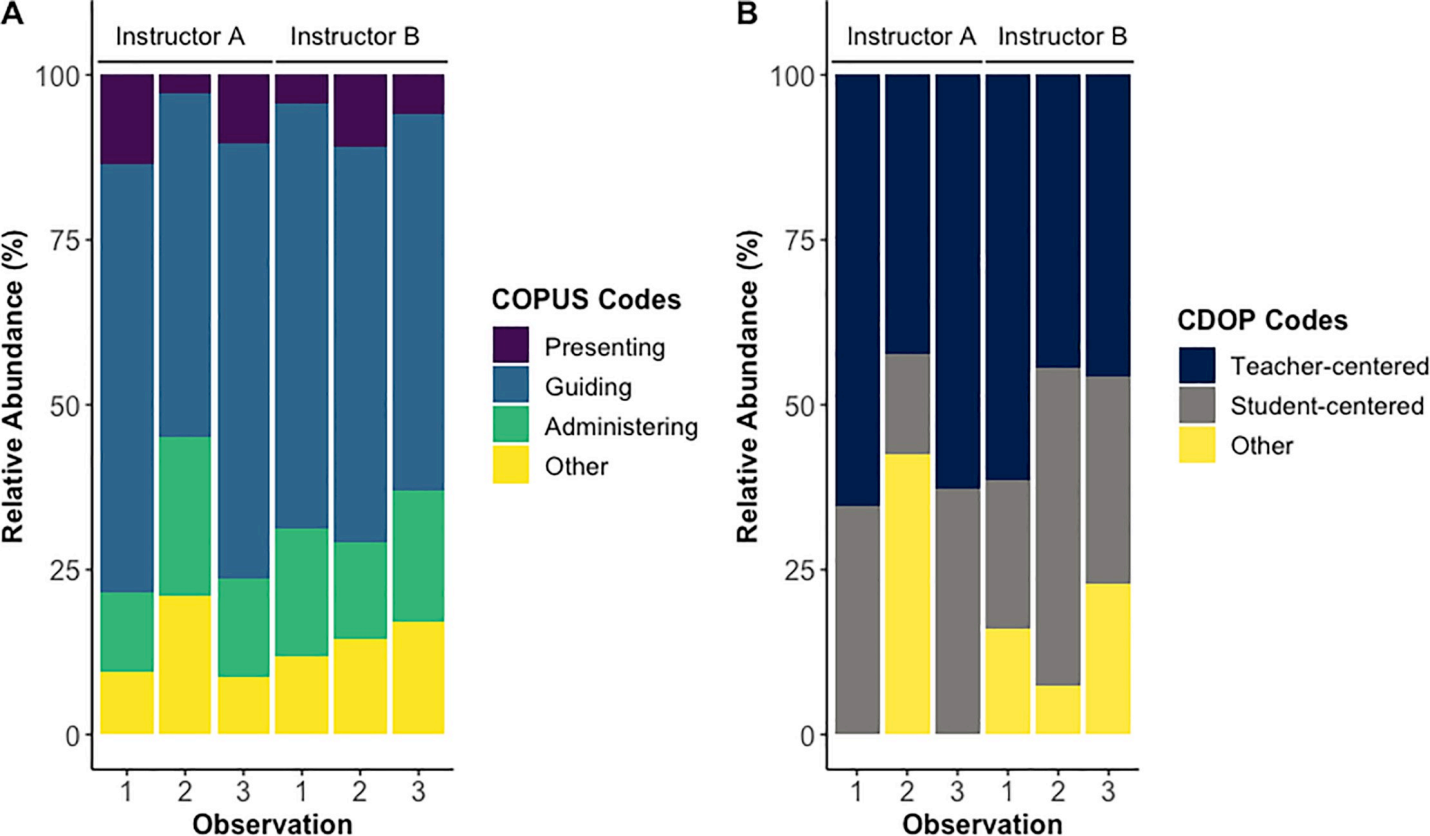
Comparison of COPUS and CDOP results between two instructors teaching in Active Learning Environments.

On average, both Instructors A and B spent about 60% of their class time *guiding* students in active learning tasks as measured by COPUS (Fig 4A), but they spent more time using *teacher-centered* discourse moves than *student-centered* and *no content discourse* ones as measured by CDOP (Fig 4B). When looking at the full class session surrounding the sample excerpts described above (i.e., Instructor A: observation 3, Instructor B: observation 2), Instructor A spent more time *guiding* students in active learning tasks (Fig 4A), but Instructor B used more *student-centered* and less *teacher-centered* discourse than instructor A (Fig 4B). These preliminary analyses indicate that the CDOP can distinguish differences in TDMs used by instructors, even in equivalently highly engaged classrooms as measured by COPUS.

**Fig 5:**
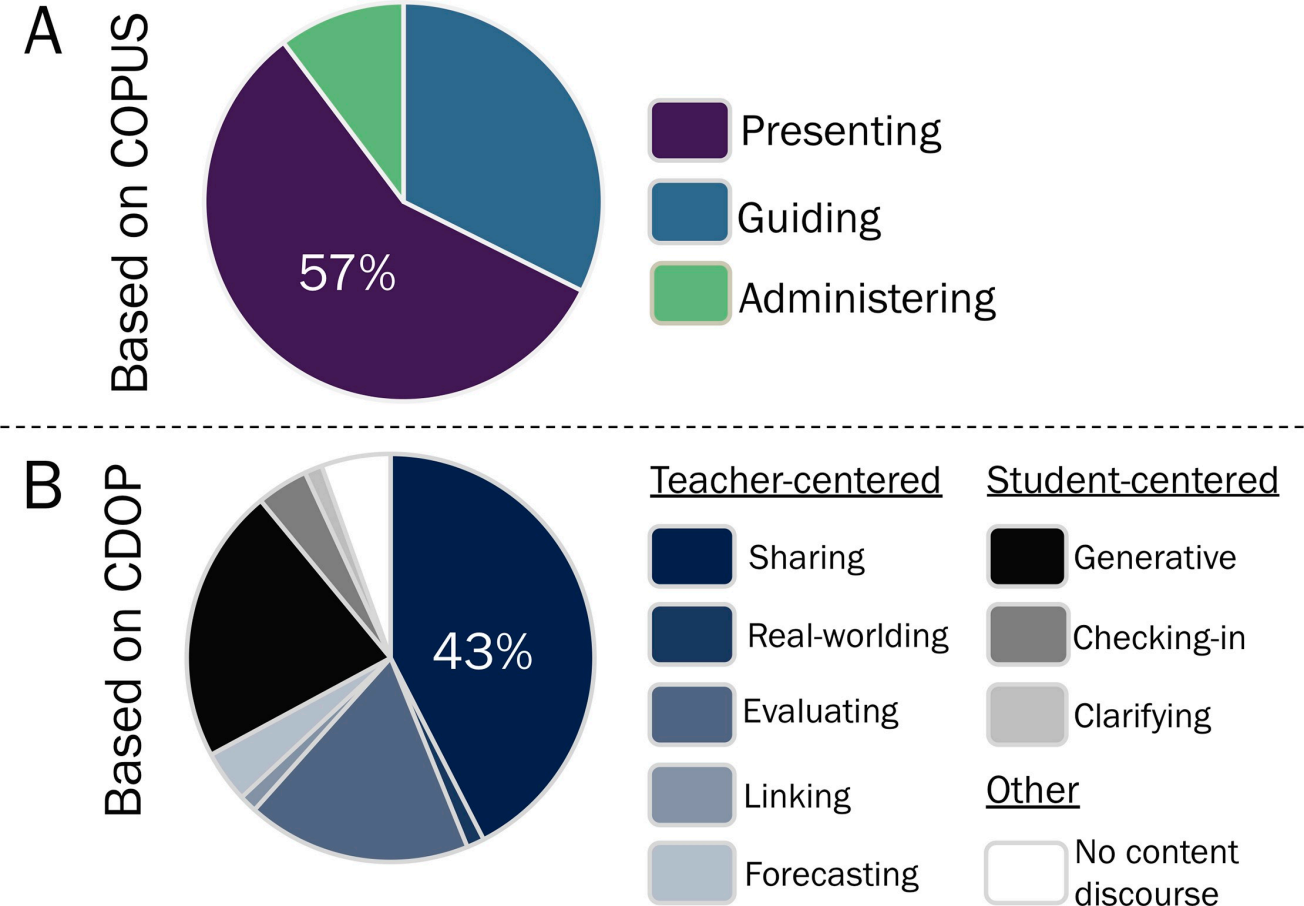
Sample CDOP results of an instructor teaching in a traditional lecture classroom.

For one class session, Instructor C spent about 57% of their class session *presenting* information to students as measured by COPUS (Fig 5A). Also, this instructor used a total of 8 discourse moves as measured by the CDOP, with information *sharing* being the most frequent (43%) followed by *generative* (22%), *evaluating* (18%,) *no content discourse* (6%*)*, *checking-in* (4%), and *forecasting* (4%), and *linking* (1%), 1% *real-worlding* (1%), and *clarifying* (1%) (Fig 5B). Five out of eight of these CDOP codes are teacher-centric and describe activities that often happen during traditional lectures (i.e., activities that are mainly proxy for content delivery). These results suggest that the CDOP can measure TDMs used by instructors than are mostly lecturing (i.e., spending most of their class *presenting* information as measured by COPUS) in addition to those that are mostly using active learning strategies (i.e., spending most of their class *guiding* students learning as measured by COPUS). See Tables A and B in S6 File for COPUS and CDOP data and results.

### Reliability and validity

We used a two-stage process to establish IRR of the CDOP. First, we qualitatively examined the TDM coding of class transcripts (*n* = 6). Cohen’s kappa scores between coder pairs ranged from 0.51 to 0.78, with an average kappa score of 0.64 among all coders (CI: 0.51–0.71, SE: 0.067; S1 File), suggesting substantial agreement among the coders according to Landis and Koch [47]. Second, we quantitatively coded audio class recordings using the CDOP matrix (*n* = 13). We first used the CDOP matrix to measure the discourse behavior of the six highest-active engagement instructors in our sample as determined by COPUS analysis. Using this subset of our overall data, the kappa scores between coder pairs ranged from 0.69 to 0.86, with an average kappa score of 0.80 (CI: 0.73–0.87, SE: 0.0364; S1 File). Once we were satisfied with the outcome of this analysis, we extended our CDOP analysis to include the remaining recordings (*n* = 7). Using CDOP, we reached high average IRR between coder pairs for all 13 instructors (κ = 0.75; CI: 0.68–0.82, SE: 0.036; S1 File). The mean kappa values we obtained when using CDOP to code class audio recordings indicate substantial to excellent agreement.

We established face and content validity of CDOP through expert panel evaluation of the instrument. On a 3-point Likert scale of agree to disagree, the panelists strongly agreed that CDOP adequately measures TDMs. The average CVI was 0.90 (S2 File), which is higher than the recommended cutoff of 0.80 for new measures [51], suggesting there was excellent expert agreement on the validity of CDOP as an instrument that can measure TDMs. Some of the panelists provided feedback that improved the operational definitions of the codes as well as helped cluster the codes which increased the functional utility of the instrument. These recommendations were incorporated into the final version of the instrument reported here.

## Discussion

Active-engagement instruction transforms the nature of student-teacher interactions, forcing instructors to constantly adjust their teaching practices to facilitate ensuing classroom discourse [9, 33]. Dialogical teaching plays a critical role in promoting an active, collaborative and cognitively-engaging learning experience for all students [21]. Therefore, we developed and validated a new instrument, CDOP, which can reliably quantify TDMs from observational data in undergraduate STEM learning environments. The CDOP coding scheme is made up of a total of 17 codes: 15 codes that capture TDMs and two more codes that can be used to either identify *other* or *no content discourse* moves. These codes identify aspects of classroom discourse and provide descriptive accounts of teacher discourse behaviors. Given the descriptive nature of the CDOP coding scheme, there is no a priori threshold or external criteria for determining an acceptable number of discursive moves. Rather, the aim of the CDOP is to provide a fine-grained detail of what TDMs are occurring in the observed classroom at the moment of observation. Therefore, the development of tools, such as CDOP, are essential for developing a nuanced understanding of how instructors facilitate student learning when the learning environment results in increased incidences of student-teacher interactions.

### Quantifying TDMs from observational data

The CDOP coding scheme identifies TDMs from classroom observational data (Table 2), while the CDOP matrix allows for efficient recording of TDMs in 2-minute time periods over the course of a class session (Fig 1). Additionally, the CDOP matrix permits tabulating the frequencies at which specific TDMs occur and making inferences about the quality of teacher’s discourse behaviors. For instance, if an instructor uses only a few CDOP codes, including the teacher-centric CDOP codes *sharing* and *evaluating* and the student-centric CDOP code *generative*, then that would suggest that the instructor is mostly using the IRE discourse pattern. However, if an instructor uses TDMs that exhibit a diversity of CDOP codes, especially student-centric ones like *explaining* or *challenging*, then there is evidence that the instructor is engaging in dialogical discourse approaches, such as the IRF discourse pattern, in their classroom.

This type of information can be used to improve how faculty orchestrate classroom discussions, especially during small group interactions. Our preliminary findings suggest that while two instructors might both highly interact with their students, one might use more *teacher-centered* TDMs than the other (Fig 4). While we have not directly tested if the more *student-centered* TDMs are more efficacious in supporting student learning gains, previous studies provide strong evidence that instructional strategies engaging students in constructive and interactive tasks are more effective than simply “being active” (e.g., [1]).

The CDOP was intentionally designed so that an observer can simply document the TDMs occurring without making holistic judgements about the instructional strategies employed by the observer. Specifically, the data collected using the CDOP matrix can be used to inform classroom instructional practices without evaluating or passing judgment on the instructional strategies that are used by the instructor (i.e., small group learning, whole class discussions, interactive lecturing, inquiry-based activities, etc.). Within this instrument, the focus becomes how an instructor orchestrates classroom discourse and documenting its various forms will empower faculty to become more aware of their own teaching practices.

Consistent with the communicative approach proposed by Mortimer and Scott [52], we note that the CDOP codes capture a spectrum of discourse behaviors as follows: 1) *sharing*, *real-worlding*, *linking* and *forecasting* indicate authoritative or instructor-driven, non-interactive discourse behavior. When making these moves, the only voice present in the discourse is that of the instructor. These codes are mostly likely to be observed in classrooms characterized by didactic lecturing; 2) *evaluating*, *generative*, and *checking-in* are similarly instructor-driven, but involve the instructor interacting with their students. These codes capture discourse behaviors in which an instructor engages students in conversation, but does not necessarily provide feedback and are most likely observed in classrooms characterized by what Stains, Harshman [12] call *interactive lectures*; and 3) *constructing*, *connecting*, *contextualizing*, *representing*, *clarifying*, *requesting*, *explaining*, and *challenging* all involve students talking and instructors providing feedback, and therefore, indicate dialogic discourse. These codes refer to situations in which the instructor not only asks students to talk about content, but that there’s some indication that the instructor listens and responds to the student talk. As such, these eight codes are most likely observed in classrooms characterized by moderate to high active engagement instruction. In general, to ensure appropriate use of CDOP, we recommend that all new observers obtain appropriate training of instrument before using it.

### CDOP observer training guide

We had high agreement (Cohen’s κ = 0.80) across multiple coder pairs, suggesting that with appropriate training new observers can use the CDOP in a similar manner. To this end, we have developed an observer training guide that allows observers to reliability characterize TDMs in undergraduate STEM learning environments. The guide contains the CDOP coding scheme (S3 File), CDOP matrix (S4 File), instruction, timing, and tips for observer training and optional video resources (S5 File).

### Limitations and future directions

Although we demonstrated internal validity of the CDOP through face and content validity, a limitation of our study is the lack of external validity, which is the degree to which the CDOP results from our sample classrooms can be generalized to other undergraduate STEM classrooms [53]. We are currently preparing a subsequent paper that will contain the CDOP results of additional instructors across multiple institutions teaching in undergraduate STEM classrooms across the United States for external validation. However, additional research groups should further validate the tool for use in other contexts.

Additionally, CDOP does not measure student discourse moves (SDMs) or the specific conversational strategies used by students to develop their content knowledge. In future studies, it would be interesting to analyze the types of SDMs used in response to TDMs in undergraduate STEM learning environments. Moreover, the Differentiated Overt Learning Activities (DOLA) framework proposed by Chi and Wylie [1] can be used to detect the degree in which the various TDMs invoke different levels of student cognitive engagement. Therefore, we plan to use the DOLA framework to categorize TDMs and determine what levels of cognitive engagement they reveal among the students.

One particular limitation of the CDOP is that it only focuses on the performative aspects of teaching–i.e., how instructors interact with students and responds to them “in the moment”–but it does not examine the design elements of teaching–i.e., how instructors create the learning environment, choose content and activities, etc. Given the complexity of classroom teaching and the focus of the CDOP on the specific conversational strategies used by instructors to foster the development of ideas in the classroom, it may be important to pair it with other classroom observational protocols, such as RTOP or COPUS, to get an holistic understanding of what is happening in a classroom. For example, if one’s interest is understanding the amount of time they spent on mainly lecture, using interactive lecturing, or utilize cooperative learning approaches, then COPUS would be a better tool to capture those classroom behaviors. The goal of CDOP is to characterize mainly the communicative approaches happening in the classroom and the student-teacher interactions regardless of the nature of the learning environment (traditional or active learning). Additionally, while an instructor may be interacting with a given student or group, other students or groups may be engaged in student-student discourse, necessitating the use of CDOP with other tools to measure student discourse in order to develop a more holistic picture.

Finally, the goal of understanding classroom discourse is to examine how different instructional strategies effect student learning outcomes. Thus, future research should examine the relationship between various TDMs in undergraduate STEM learning environments and student learning outcomes as measured by pre-posttest tools. For example, the research question could be: Is there a differential impact of various TDMs on student learning gains?

## Conclusions

The present study reported the development and validity analysis of an instrument, the Classroom Discourse Observation Protocol (CDOP), which reliably quantifies teacher discourse moves (TDMs) in undergraduate STEM learning environments. TDMs are essential features of classroom learning, particularly in active learning environments that increase the incidences of student-teacher interactions. The CDOP coding scheme and the CDOP matrix described in the paper allow observers to capture, on a 2-minute interval basis, the frequency of TDMs occurring over the course of a class period. We found high inter-rater reliability among multiple coders when using the CDOP (Cohen’s Kappa values of 0.75 and 0.80). We also found preliminarily that the CDOP is able to detect subtle differences among instructors who are otherwise using similar active learning strategies. Thus, the development of CDOP profiles makes it possible to explore how different faculty orchestrate classroom discourse. This suggests CDOP can be used as a professional development tool to explore instructional practices that are the most effective when teaching in undergraduate STEM learning environments.

## Supporting information

S1 File Inter-rater reliability calculations among coder pairs. (PDF)

S2 File Expert rating of CDOP item content validity. (PDF)

S3 File CDOP code book. (PDF)

S4 File Complete CDOP matrix. (PDF)

S5 File CDOP observer training guide. (DOCX)

S6 File Manuscript data and results. (XLSX)

## References

[1] Chi MTH, Wylie R. The ICAP framework: Linking cognitive engagement to active learning outcomes. Educational Psychologist. 2014;49(4):219–43. 10.1080/00461520.2014.965823

[2] Baepler PM, Walker JD, Brooks DC, Saichaie K, Petersen C. A guide to teaching in the active learning classroom: history, research, and practice. Sterling, Virginia: Stylus Publishing; 2016. 269 p.

[3] National Research Council. Discipline-based education research: understanding and improving learning in undergraduate science and engineering. Washington, DC: The National Academies Press, 2012 s.

[4] Association of American Universities. Progress toward achieving systemic change: Five-year status report on the AAU Undergraduate STEM Education Initiative. Washington, DC.: 2017.

[5] Hora MT, Ferrare JJ. Instructional systems of practice: A multidimensional analysis of math and science undergraduate course planning and classroom teaching. Journal of the Learning Sciences. 2013;22(2):212–57. 10.1080/10508406.2012.729767

[6] Freeman S, Eddy SL, McDonough M, Smith MK, Okoroafor N, Jordt H, et al Active learning increases student performance in science, engineering, and mathematics. Proceedings of the National Academy of Sciences. 2014;111(23):8410–5. 10.1073/pnas.1319030111 24821756 PMC4060654

[7] Dolan EL. Biology education research 2.0. CBE—Life Sciences Education. 2015;14(4):ed1 10.1187/cbe.15-11-0229 . 26628560 PMC4710408

[8] Michaels S, Sohmer RE, O’Connor MC. Classroom discourse. Sociolinguistics: An international handbook of the science of language and society. 2004:2351–66.

[9] Walshaw M, Anthony G. The teacher’s role in classroom discourse: A review of recent research into mathematics classrooms. Review of educational research. 2008;78(3):516–51.

[10] Smith MK, Jones FHM, Gilbert SL, Wieman CE. The Classroom Observation Protocol For Undergraduate Stem (COPUS): A new instrument to characterize university STEM classroom practices. CBE-Life Sciences Education. 2013;12(4):618–27. 10.1187/cbe.13-08-0154 24297289 PMC3846513

[11] Smith MK, Vinson EL, Smith JA, Lewin JD, Stetzer MR. A campus-wide study of stem courses: New perspectives on teaching practices and perceptions. CBE-Life Sciences Education. 2014;13(4):624–35. 10.1187/cbe.14-06-0108 25452485 PMC4255349

[12] Stains M, Harshman J, Barker MK, Chasteen SV, Cole R, DeChenne-Peters SE, et al Anatomy of STEM teaching in North American universities. Science. 2018;359(6383):1468–70. 10.1126/science.aap8892 29599232 PMC6310123

[13] Michaels S, O’Connor C. Conceptualizing talk moves as tools: Professional development approaches for academically productive discussion. Socializing intelligence through talk and dialogue. 2015:347–62.

[14] Duschl R. Science education in three-part harmony: Balancing conceptual, epistemic, and social learning goals. Review of research in education. 2008;32(1):268–91.

[15] Christodoulou A, Osborne J. The science classroom as a site of epistemic talk: A case study of a teacher's attempts to teach science based on argument. Journal of Research in Science Teaching. 2014;51(10):1275–300.

[16] Ohlsson S. Learning to do and learning to understand: a lesson and a challenge for cognitive modeling. In: Reiman P, Spade H, editors. Learning in humans and machines towards an interdisciplinary learning science: Pergamon; 1996 p. 37–62.

[17] Cazden CB. Classroom discourse: The language of teaching and learning. Portsmouth, NH: Heinemann; 2001.

[18] Chapin SH, O'Connor C, Anderson NC. Classroom discussions: Using math talk to help students learn, grades K-6: Math Solutions; 2009.

[19] Herbel-Eisenmann BA, Steele MD, Cirillo M. (Developing) teacher discourse moves: A framework for professional development. Mathematics Teacher Educator. 2013;1(2):181–96.

[20] Howe C, Abedin M. Classroom dialogue: A systematic review across four decades of research. Cambridge journal of education. 2013;43(3):325–56.

[21] Alexander RJ. Towards dialogic teaching: rethinking classroom talk: Dorchester Publishing Company, Incorporated; 2008.

[22] Michaels S, O’Connor C. Talk science primer. Cambridge, MA: TERC; 2012.

[23] O'Connor C, Michaels S, Chapin S, Harbaugh AG. The silent and the vocal: participation and learning in whole-class discussion. Learning and Instruction. 2017;48:5–13.

[24] Sawada D, Piburn MD, Judson E, Turley J, Falconer K, Benford R, et al Measuring reform practices in science and mathematics classrooms: the reformed teaching observation protocol. School Science and Mathematics. 2002;102(6):245–53. 10.1111/j.1949-8594.2002.tb17883.x

[25] Wieman C, S. G, Dolan EL. The Teaching Practices Inventory: A new tool for characterizing college and university teaching in mathematics and science. CBE—Life Sciences Education. 2014;13(3):552–69. 10.1187/cbe.14-02-0023 . 25185237 PMC4152215

[26] Gee JP. How to do discourse analysis: A toolkit. London: Routledge; 2010. 224 p.

[27] Rojas-Drummond S, Torreblanca O, Pedraza H, Vélez M, Guzmán K. ‘Dialogic scaffolding’: Enhancing learning and understanding in collaborative contexts. Learning, Culture and Social Interaction. 2013;2(1):11–21.

[28] van de Pol J, Elbers E. Scaffolding student learning: A micro-analysis of teacher–student interaction. Learning, Culture and Social Interaction. 2013;2(1):32–41. 10.1016/j.lcsi.2012.12.001.

[29] Prediger S, Pöhler B. The interplay of micro- and macro-scaffolding: an empirical reconstruction for the case of an intervention on percentages. ZDM. 2015;47(7):1179–94. 10.1007/s11858-015-0723-2

[30] Strauss A, Corbin J. Basics of grounded theory methods. Beverly Hills, CA: Sage; 1990.

[31] Pimentel DS, McNeill KL. Conducting talk in secondary science classrooms: Investigating instructional moves and teachers’ beliefs. Science Education. 2013;97(3):367–94.

[32] Chin C. Teacher questioning in science classrooms: Approaches that stimulate productive thinking. Journal of Research in Science Teaching: The Official Journal of the National Association for Research in Science Teaching. 2007;44(6):815–43.

[33] Hardman J. Tutor–student interaction in seminar teaching: Implications for professional development. Active Learning in Higher Education. 2016;17(1):63–76.

[34] Warfa A-RM, Roehrig GH, Schneider JL, Nyachwaya J. Role of teacher-initiated discourses in students’ development of representational fluency in chemistry: A case study. Journal of Chemical Education. 2014;91(6):784–92. 10.1021/ed4005547

[35] Rasmussen C, Kwon O, Marrongelle K, editors. A framework for interpreting inquiry-oriented teaching. Conference on Research in Undergraduate Mathematics Education, Mission Valley, CA; 2008.

[36] Sinclair JM, Coulthard M. Towards an analysis of discourse: The English used by teachers and pupils. London: Oxford University Press; 1975.

[37] Mehan H. Learning lessons: Social organization in the classroom. Cambridge, MA: Harvard University Press; 1979. 227 p.

[38] Garton S. Speaking out of turn? Taking the initiative in teacher-fronted classroom interaction. Classroom Discourse. 2012;3(1):29–45.

[39] Krussel L, Edwards B, Springer G. The teacher's discourse moves: A framework for analyzing discourse in mathematics classrooms. School Science and Mathematics. 2004;104(7):307–12.

[40] Lidar M, Lundqvist E, Östman L. Teaching and learning in the science classroom: The interplay between teachers' epistemological moves and students' practical epistemology. Science Education. 2006;90(1):148–63. 10.1002/sce.20092

[41] Criswell BA, Rushton GT. Conceptual change, productive practices, and themata: supporting chemistry classroom talk. Journal of Chemical Education. 2012;89(10):1236–42.

[42] MacDonald R, Miller E, Lord S. Doing and Talking Science: Engaging ELs in the Discourse of the Science and Engineering Practices In: Oliveira AW, Weinburgh MH, editors. Science Teacher Preparation in Content-Based Second Language Acquisition. Cham: Springer International Publishing; 2017 p. 179–97.

[43] NGSS Lead States. Next generation science standards: For states, by states. Washington, DC: The National Academies Press; 2013.

[44] O’Connor C, Michaels S, Chapin S. Scaling down” to explore the role of talk in learning: From district intervention to controlled classroom study. Socializing intelligence through academic talk and dialogue. 2015:111–26.

[45] Seidel SB, Reggi AL, Schinske JN, Burrus LW, Tanner KD, Tomanek D. Beyond the Biology: A Systematic Investigation of Noncontent Instructor Talk in an Introductory Biology Course. CBE—Life Sciences Education. 2015;14(4):ar43 10.1187/cbe.15-03-0049 . 26582237 PMC4710404

[46] R Core Team. R: A language and environment for statistical computing. Vienna, Austria: R Foundation for Statistical Computing; 2018.

[47] Landis JR, Koch GG. The measurement of observer agreement for categorical data. Biometrics. 1977;33(1):159–74. 10.2307/2529310 843571

[48] Nunnally JC, Bernstein I. Psychometric theory: McGraw-Hill New York; 1994.

[49] Hubley AM, Zumbo BD. A dialectic on validity: Where we have been and where we are going. The Journal of General Psychology. 1996;123(3):207–15.

[50] Rubio DM, Berg-Weger M, Tebb SS, Lee ES, Rauch S. Objectifying content validity: Conducting a content validity study in social work research. Social work research. 2003;27(2):94–104.

[51] Davis LL. Instrument review: Getting the most from a panel of experts. Applied nursing research. 1992;5(4):194–7.

[52] Mortimer EF, Scott PH. Meaning Making in Secondary Science Classrooms. Maidenhead: Open University Press; 2003.

[53] Cohen L, Manion L, Morrison K. Validity and reliability Research Methods in Education. New York, NY: Routledge; 2007.

