# Supplementary material for: The Classroom Discourse Observation Protocol (CDOP): A quantitative method for characterizing teacher discourse moves in undergraduate STEM learning environments
Source: PLoS One. 2019 Jul 17;14(7):e0219019. doi: 10.1371/journal.pone.0219019 (PMC6636728; doi:10.1371/journal.pone.0219019)
Supplement: S1 File — (PDF) [file pone.0219019.s001.pdf]

Table S1.1. Average Cohen's kappa IRR scores for CDOP coders using class transcripts of six instructors

| **Instructor Code** | **Class Session** | **Coder pairs** | **No. of lines** | **Kappa** | **SE** | **Confidence Intervals** | |
|---|---|---|---|---|---|---|---|
| | | | | | | Lower | Upper |
| 100 | 16-Oct-17 | Coder 1/Coder 2 | 135 | 0.67 | 0.064 | 0.55 | 0.80 |
| 101 | 27-Sep-17 | Coder 1/Coder 2 | 84 | 0.57 | 0.089 | 0.40 | 0.75 |
| 102 | 4-Oct-17 | Coder 1/Coder 2 | 137 | 0.69 | 0.064 | 0.56 | 0.82 |
| 105 | 23-Oct-17 | Coder 1/Coder 2 | 127 | 0.78 | 0.057 | 0.67 | 0.90 |
| 108 | 17-Oct-17 | Coder 1/Coder 2 | 167 | 0.60 | 0.064 | 0.48 | 0.73 |
| 112 | 2-Oct-17 | Coder 1/Coder 2 | 235 | 0.51 | 0.061 | 0.39 | 0.39 |
| | | **Total no. of lines** | 885 | 0.64 | 0.067 | 0.51 | 0.73 |

Table S1.2. Average Cohen's kappa IRR scores for CDOP coders using audio class recordings of six instructors.

| **Instructor Code** | **Class Session** | **Coder pairs** | **No. of lines** | **Kappa** | **SE** | **Confidence Intervals** | |
|---|---|---|---|---|---|---|---|
| | | | | | | Lower | Upper |
| 100 | 16-Oct-17 | Coder 1/Coder 2 | 110 | 0.69 | 0.035 | 0.63 | 0.76 |
| 101 | 27-Sep-17 | Coder 1/Coder 2 | 108 | 0.79 | 0.033 | 0.73 | 0.86 |
| 102 | 4-Oct-17 | Coder 1/Coder 2 | 90 | 0.86 | 0.030 | 0.80 | 0.91 |
| 105 | 23-Oct-17 | Coder 1/Coder 2 | 50 | 0.79 | 0.046 | 0.70 | 0.88 |
| 108 | 17-Oct-17 | Coder 1/Coder 2 | 78 | 0.85 | 0.031 | 0.79 | 0.92 |
| 112 | 2-Oct-17 | Coder 1/Coder 2 | 74 | 0.81 | 0.030 | 0.76 | 0.87 |
| | | Total minutes | 510 | | | | |
| | | Total hours | 8.5 | 0.80 | 0.034 | 0.73 | 0.87 |

Table S1.3. Average Cohen's kappa IRR scores for CDOP coders using audio class recordings of 13 instructors.

| **Instructor Code** | **Class Session** | **Coder pairs** | **No. of lines** | **Kappa** | **SE** | **Confidence Intervals** | |
|---|---|---|---|---|---|---|---|
| | | | | | | Lower | Upper |
| 100 | 16-Oct-17 | Coder 2/Coder 4 | 110 | 0.58 | 0.039 | 0.51 | 0.66 |
| 101 | 27-Sep-17 | Coder 2/Coder 4 | 108 | 0.79 | 0.035 | 0.72 | 0.86 |
| 102 | 4-Oct-17 | Coder 1/Coder 3 | 90 | 0.75 | 0.038 | 0.68 | 0.83 |
| 103 | 5-Oct-17 | Coder 2/Coder 3 | 76 | 0.77 | 0.040 | 0.69 | 0.84 |
| 104 | 10-Oct-17 | Coder 2/Coder 4 | 80 | 0.72 | 0.040 | 0.64 | 0.79 |
| 105 | 23-Oct-17 | Coder 1/Coder 4 | 50 | 0.77 | 0.048 | 0.67 | 0.86 |
| 106 | 12-Oct-17 | Coder 1/Coder 3 | 74 | 0.61 | 0.043 | 0.52 | 0.69 |
| 108 | 17-Oct-17 | Coder 2/Coder 4 | 78 | 0.76 | 0.040 | 0.68 | 0.83 |
| 109 | 14-Nov-17 | Coder 2/Coder 3 | 108 | 0.93 | 0.019 | 0.89 | 0.96 |
| 110 | 16-Oct-17 | Coder 2/Coder 4 | 114 | 0.79 | 0.032 | 0.73 | 0.85 |
| 111 | 28-Sep-17 | Coder 1/Coder 4 | 76 | 0.74 | 0.040 | 0.66 | 0.82 |
| 112 | 2-Oct-17 | Coder 1/Coder 3 | 74 | 0.66 | 0.039 | 0.58 | 0.73 |
| 113 | 26-Sep-17 | Coder 1/Coder 3 | 116 | 0.90 | 0.019 | 0.86 | 0.94 |
| | | Total minutes | 1154 | | | | |
| | | Total hours | 19.2 | **0.75** | **0.036** | 0.68 | 0.82 |
