# Supplementary material for: The Classroom Discourse Observation Protocol (CDOP): A quantitative method for characterizing teacher discourse moves in undergraduate STEM learning environments
Source: PLoS One. 2019 Jul 17;14(7):e0219019. doi: 10.1371/journal.pone.0219019 (PMC6636728; doi:10.1371/journal.pone.0219019)
Supplement: S2 File — (PDF) [file pone.0219019.s002.pdf]

Table S2. CDOP items as rated by experts for content validity

Items	Experts 1	2	3	4	5	CVI
Representativeness	3	3	3	3	3	1.00
Clarity and overall structure	3	3	1	3	3	0.80
Tool usefulness	3	3	3	3	3	1.00
Comprehensiveness	3	1	3	3	3	0.80
Average Content Validity Indext (CVI):						0.90
