# Supplementary material for: The Classroom Discourse Observation Protocol (CDOP): A quantitative method for characterizing teacher discourse moves in undergraduate STEM learning environments
Source: PLoS One. 2019 Jul 17;14(7):e0219019. doi: 10.1371/journal.pone.0219019 (PMC6636728; doi:10.1371/journal.pone.0219019)
Supplement: S3 File — (PDF) [file pone.0219019.s003.pdf]

Classroom Discourse Observation Protocol (CDOP) for Undergraduate STEM Classrooms

This protocol allows observers to reliably characterize the specific conversational strategies used by instructors to foster students development of content knowledge.

**1. Teacher-centered: Instructor is talking about content.**		
**Codes**	**Description**	**Dialogue**
**sharing**	Instructor shares information, answers student question, or provides instructions for finding the solution	**Instructor: Just think of, kind of, chromatid pairs, sister chromatid paired, it's a little easier to think of the numbers.**
**real-worlding**	Instructor relates idea to conventional knowledge, broader perspective, and instructor's or student's personal experiences	**Instructor: Successful genotypes-look around the room. Nothing but winner in this room, right? We have all made it to reproductive age.**
**evaluating**	Instructor repeats, accepts and/or rejects student's response, or acknowledges that they don't know the answer to a student's question	Student: And then in the first case, it would be one chance times one chance which is still one sixteenth. **Instructor: Right.**
**linking**	Instructor associates past topic to current topic	Student: You don't have a bigger potential as well because there's more connections, there's more access to the axon terminals? **Instructor: Well, remember, we had that summation of action potentials. We had an action potential and we had the nodes and it could split off.**
**forecasting**	Instructor associates current topic to future topic	**Instructor: You're going to do something in lab actually focused on human population and population growth.**

**2. Student-centered: Instructor asks student to talk about content.**		
**Codes**	**Description**	**Dialogue**
**generative**	Instructor asks student to recall facts, and basic concepts, or related information	**Instructor: Those come together in fertilization to make a zygote, right? Student: Yes.**
**checking-in**	Instructor asks student if they have a question or need clarification	**Instructor: Does that make sense?; Do you have any questions?; How's it going?; Are we good?**
**clarifying**	Instructor asks student to elaborate on condensed, cryptic, or inexplicit statement	**Instructor: Can you say more about that? What do you mean by that? Can you give an example?**
**connecting**	Instructor asks student to associate past topic to current topic	**Instructor: Costs of sex that haven't been mentioned plus what we've been talking about for the last week.** Student: Is it overpopulation?
**contextualizing**	Instructor asks students to relate idea to conventional knowledge, broader perspective, and their personal experiences	**Instructor: Anyone have an example that they really want to hear about/talk about** (referring to student responses to finding analogies between cell processes and common household items)?
**representing**	Instructor asks student to create a visual or mathematical representation of content	**Instructor: Think about how you could draw that out, too.**
**constructing**	Instructor asks students to build knowledge by interpreting and/or making judgments based on evidence, data, and/or model	**Instructor: In your own words, what is your conclusion when you look at those data?**
**requesting**	Instructor asks student to justify or explain their reasoning	**Instructor: I'm liking what I see but explain it to me** (referring to student whiteboard work calculating the number of fertilization events that produce a specific offspring).
**explaining**	Instructor asks student to explain reasoning to other students	**Instructor: Can you explain your work to everybody else at your table so that they can figure that out?**
**challenging**	Instructor asks student to evaluate another student's idea	**Instructor: Cost of sex?** Student: Pregnancy. **Instructor: I acknowledge that it's a good point, and why is there a problem with calling pregnancy a cost evolutionarily?**

**3. Other**	
**Codes**	**Description**
**no content discourse**	Instructor is not talking or asking students to talk about content
**other**	TDM not described by these codes
