# Supplementary material for: The Classroom Discourse Observation Protocol (CDOP): A quantitative method for characterizing teacher discourse moves in undergraduate STEM learning environments
Source: PLoS One. 2019 Jul 17;14(7):e0219019. doi: 10.1371/journal.pone.0219019 (PMC6636728; doi:10.1371/journal.pone.0219019)
Supplement: S5 File — (DOCX) [file pone.0219019.s005.docx]

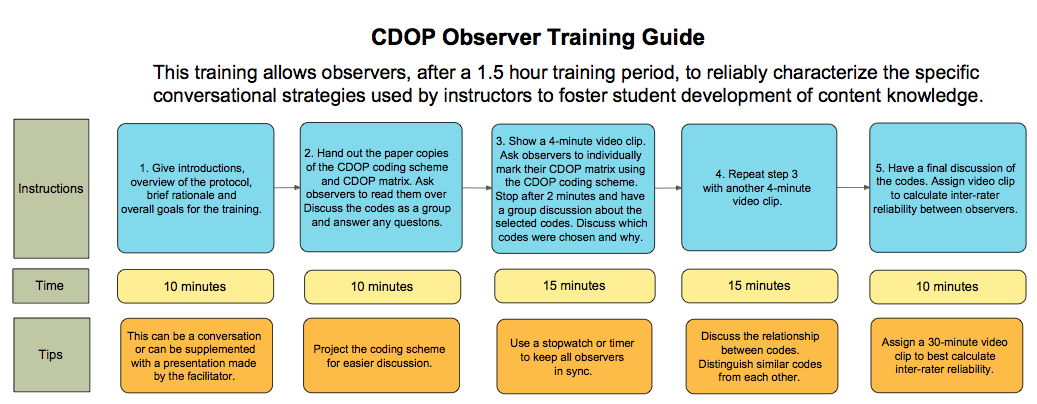


Below are videos that could be used for training purpose.

| **Video** | **Video Title** | **Link** |
| --- | --- | --- |
| 1 | REALISE Videos 2 (approx. 5 min) | <https://www.youtube.com/watch?v=iDs1RPSpXhE> |
| 2 | REALISE Videos 5 (approx. 8.5 min) | <https://www.youtube.com/watch?v=sLRgP734J7g> |
| 3 | Interactive teaching (approx. 8.5 min) | <https://www.youtube.com/watch?v=wont2v_LZ1E> |
| 4 | Interactive lecturing (approx. 46 min) | [https://ocw.mit.edu/courses/chemistry/5-111-principles-of-chemical-science-fall-2008/video-lectures/lecture-19](https://ocw.mit.edu/courses/chemistry/5-111-principles-of-chemical-science-fall-2008/video-lectures/lecture-19/)/ |
| 5 | REALISE Videos 3 (approx. 15 min) | <https://www.youtube.com/watch?v=ZlqoEUZQBoY> |
| 6 | REALISE Videos 6 (approx. 5 min) | <https://www.youtube.com/watch?v=dFtpertlh9E> |
| 7 | REALISE Videos 7 (approx. 4.5 min) | <https://www.youtube.com/watch?v=W0SC5ovGdOM> |
| 8 | All REALISE Videos | <https://seercenter.uga.edu/realisevideos_immerse/> |
